# Epidermal growth factor receptor (*EGFR*) T790M mutation identified in plasma indicates failure sites and predicts clinical prognosis in non-small cell lung cancer progression during first-generation tyrosine kinase inhibitor therapy: a prospective observational study

**DOI:** 10.1186/s40880-018-0303-2

**Published:** 2018-05-22

**Authors:** Shirong Zhang, Lucheng Zhu, Bing Xia, Enguo Chen, Qiong Zhao, Xiaochen Zhang, Xueqin Chen, Xufeng Chen, Shenglin Ma

**Affiliations:** 1 Center for Translational Medicine, Hangzhou First People’s Hospital, Nanjing Medical University, No. 261 Huansha Road, Shangcheng District, Hangzhou, 310006 Zhejiang China; 2 Department of Oncology, Hangzhou First People’s Hospital, Nanjing Medical University, Hangzhou, 310006 Zhejiang China; 3 Department of Oncology, Hangzhou Cancer Hospital, Hangzhou, 310000 Zhejiang China; 4 Department of Respiratory, Sir Run Run Shaw Hospital, School of Medicine, Zhejiang University, Hangzhou, 310020 Zhejiang China; 5 Department of Oncology, the First Affiliated Hospital, Zhejiang University, Hangzhou, 310006 Zhejiang China; 6 Department of Cancer Genetics and Epigenetics, City of Hope National Medical Center, Duarte, CA 91010 USA

**Keywords:** Non-small cell lung cancer, Epidermal growth factor receptor, T790M, ctDNA, Failure sites

## Abstract

**Introduction:**

Plasma circulating tumor DNA (ctDNA) is an ideal approach to detecting the epidermal growth factor receptor (*EGFR*) T790M mutation, which is a major mechanism of resistance to first-generation EGFR-tyrosine kinase inhibitor (TKI) therapy. The present study aimed to explore the association of ctDNA-identified T790M mutation with disease failure sites and clinical prognosis in non-small cell lung cancer (NSCLC) patients.

**Methods:**

Patients who progressed on first-generation TKIs were categorized into failure site groups of chest limited (CF), brain limited (BF) and other (OF). Amplification refractory mutation system (ARMS) and droplet digital PCR (ddPCR) were used to identify the T790M mutation in ctDNA. Prognosis was analyzed with Kaplan–Meier methods.

**Results:**

Overall concordance between the two methods was 78.3%. According to both ARMS and ddPCR, patients in the OF group had a significantly higher rate of T790M mutation than did patients in the BF and CF groups (*P *< 0.001), and a significantly higher T790M mutation rate was also observed in OF-group patients than in those in the CF and BF groups (*P *< 0.001). AZD9291 was found to be an excellent treatment option and yielded the longest survival for T790M+ patients in all groups who had progressed on EGFR-TKIs; for other treatments, the prognosis of T790M− patient subgroups varied.

**Conclusions:**

The present study demonstrates that T790M mutation in ctDNA is associated with failure sites for NSCLC patients after EGFR-TKI therapy and indicates that both failure site and T790M mutational status greatly influence treatment selection and prognosis.

**Electronic supplementary material:**

The online version of this article (10.1186/s40880-018-0303-2) contains supplementary material, which is available to authorized users.

## Introduction

Approximately 85% of lung cancers are classified as non-small cell lung cancer (NSCLC). Among these patients, approximately 10% in the US and 35% in East Asia harbor epidermal growth factor receptor (*EGFR*) mutations in the tyrosine kinase domain (exons 18–21). *EGFR* mutations are usually heterozygous with amplification in the mutant allele [1]. Approximately 90% of these mutations are the exon 19 deletion (19del) or exon 21 L858R point mutation [2], which can increase the activity of EGFR kinase and activate downstream pro-survival signaling pathways [3]. EGFR-tyrosine kinase inhibitors (EGFR-TKIs) are highly effective clinical agents for NSCLC patients with *EGFR* mutations and can result in an objective response rate (ORR) of approximately 70% and prolong progression-free survival (PFS) to 8–13 months [4–9]. Although the initial response to EGFR-TKI therapy is rapid, prolonging ORR and PFS, the favorable therapeutic effects are not maintained due to acquisition of resistance to inhibitors after a median response duration of 9–13 months [10]. The most common mutation associated with acquired resistance is T790M, a secondary point mutation located in exon 20 that results in the substitution of methionine for threonine at position 790. The *EGFR* T790M mutation is present in over 50% of NSCLC patients with EGFR-TKI resistance [10]. Other molecular mechanisms for EGFR-TKI resistance include hepatocyte growth factor receptor (c-MET) amplification [11], erbb2 receptor tyrosine kinase 2 (HER2) and phosphatidylinositol-4,5-bisphosphate 3-kinase catalytic subunit alpha (PIK3CA) mutation [12], BCL2-like 11 (BIM) polymorphism [13], and transformation to small cell lung cancer [14].

Recurrence or lesion growth in advanced NSCLC patients can be found in the lung, the mediastinum, distant organs such as the liver and bone, or the central nervous system (CNS), requiring different treatment. For example, isolated or metastatic lesions in the lung, mediastinum and CNS may benefit from radiation or other local therapies, whereas distant lesions need to be treated with chemotherapy or third-generation EGFR-TKIs (AZD9291). Compared to those with other resistance mechanisms, patients with T790M mutation after EGFR-TKI treatment may present distinct modes of recurrence or progression. A previous study showed that the presence of T790M mutation in patients with acquired resistance to EGFR-TKIs was associated with a favorable prognosis and that these patients had longer PFS and overall survival (OS) than did those who acquired resistance via other mechanisms [15]. A preclinical model also revealed indolent growth for cells with acquired T790M mutation [16–20]. Another study reported that T790M mutation is more readily detected in the plasma of patients with extra-thoracic metastatic disease (M1b) than in the plasma of patients with intra-thoracic lesions (M1a/M0); thus, patients with T790M mutation in circulating tumor DNA (ctDNA) have a high likelihood of developing distant metastases [21]. In addition, T790M mutation in ctDNA is associated with a significantly shorter OS than is ctDNA negative for the mutation [22]. Therefore, T790M mutation identified in ctDNA may serve as a marker for clinical outcomes and failure after EGFR-TKI therapy.

Modes of clinical failure for EGFR-TKI therapy are typically based on the duration of disease control and evaluation of the tumor burden and clinical symptoms [23]. However, the relationship between failure sites for EGFR-TKIs and the T790M mutational status have remained unclear, and this issue needs to be resolved. Although Carrera et al. [12] reported no significant difference in the distribution of *EGFR* T790M mutation within various failure sites after TKI therapy, the recent study referred to above showed that T790M mutation was more readily detected in the plasma of M1b patients than in that of M1a/M0 patients [21]. Moreover, ctDNA-identified T790M mutation is more frequently observed in patients with new lesions or distant metastasis than in those with local lesions, indicating the prognostic value of T790M mutation with regard to tumor progression and metastasis [24]. Nevertheless, the relationship between failure sites of TKI treatment and T790M mutation in ctDNA has yet to be clarified. Therefore, it is necessary to investigate the potential mechanisms and roles of the T790M mutation in NSCLC patients who exhibit different failure sites after treatment with EGFR-TKIs.

Detection of ctDNA-based mutations is very promising due to several notable advantages, including the non-invasive nature of the assay, the accessibility of samples and the potential for repeated sampling, especially following progression after first-line TKI therapy. The detection rate of T790M mutation in ctDNA from NSCLC patients with acquired resistance to TKIs ranges from 30–50% via qualitative assays such as BEAMing (beads, emulsion, amplification, and magnetics) digital PCR [25], droplet digital PCR (ddPCR) [26], and next-generation sequencing (NGS)-based methods [26]. Although several studies have assessed the prognostic value of T790M mutation identified in ctDNA [20, 22, 27], associations of failure sites with TKI treatment and T790M mutation in ctDNA have not been explored. Hence, the present study aimed to determine whether the frequency and abundance of T790M mutation in ctDNA indicates failure sites and enables analysis of the prognostic value of these mutations in patients with disease failure sites after the acquisition of resistance to first-generation EGFR-TKI treatment.

## Patients and methods

### Study population

This prospective, observational, multi-institutional study was performed between March 2015 and March 2016. The protocol was approved by the Institutional Review Board of Hangzhou First People’s Hospital (No. HZFH CA15-02). All patients signed informed consent. The present study was registered in clinicaltrials.gov (NCT02418234). All experiments were performed in accordance with relevant guidelines and regulations. Patients were considered eligible and enrolled in the present study if they met the following criteria: (1) presence of histologically confirmed stage III/IV NSCLC; (2) presence of activating *EGFR* mutations (G719A/C/S/X; exon 19 insertion (Ins)/Del; L858R; L861Q; or 20Ins) or response to treatment or durable stable disease (≥ 6 months) to EGFR-TKIs (erlotinib, gefitinib and icotinib) followed by progression during TKI treatment; (3) presence of tumors clinically resistant to first-generation EGFR-TKIs according to Jackman’s criteria [28]; (4) samples collected before all treatment; and (5) available data corresponding to clinical features, best TKI response, site(s) of failure, and duration of response. Follow-up was performed every 3 months via telephone calls.

### Definition of disease failure sites

To analyze failure sites, two senior thoracic radiologists reviewed radiological images to evaluate disease progression at the original (primary and metastatic) or new site(s). Patients were categorized into three groups according to the following failure sites: (1) chest limited (CF), defined as progressive disease (PD) that was limited to the chest in lung/pleural tissues and lymph nodes, with no evidence of progression beyond the chest; (2) brain limited (BF), defined as PD in an previously existing site or a new site of metastatic disease in the brain, with no evidence of extracranial progression; and (3) other (OF), defined as PD at other distant sites or multiple sites including the chest and intracranial region.

### Sample collection and DNA extraction

Blood samples were collected within 14 days after the development of TKI resistance, as assessed by the physician according to the Jackman criteria [28]. Approximately 10–15 mL of peripheral blood was collected into a cell-free DNA protection vacuum tube (AmoyDx, Xiamen, Fujian, China) containing a cell-free DNA protection reagent that promotes DNA stability for 7 days at 4–25 °C. Blood samples were transported to the Center for Translational Medicine of Hangzhou First People’s Hospital within 36 h for further processing. For DNA extraction, the blood samples were centrifuged at 2500×*g* for 10 min at 4 °C. The supernatant was transferred to a new tube and centrifuged at 15,800×*g* for 15 min at 4 °C. The supernatant (plasma) was stored at −80 °C. Cell-free DNA from 1.5 mL plasma was extracted with a QIAamp Circulating Nucleic Acid kit according to the manufacturer’s instructions (Qiagen, Hilden, Germany).

### Detection of *EGFR* mutations in plasma ctDNA by ARMS

*EGFR* mutations in plasma ctDNA were determined by using the ADx-ARMS kit (AmoyDx), and all experiments and genotype calling were performed according to the manufacturer’s instructions [29].

### ddPCR assays for *EGFR* T790M mutation determination

Recently, ddPCR has become a well-developed method for rapidly and quantitatively assessing *EGFR* mutations with high specificity and sensitivity [22, 26, 30–33]. *EGFR* T790M mutational status was determined by ddPCR with AmoyDx EGFR Exon 20 T790M Mutation Detection Kit. The principle of ddPCR for T790M mutation testing is shown in Additional file 1: Figure S1. Briefly, two probes with one nucleotide difference that target the mutated region were labeled with carboxyfluorescein (FAM) and green fluorescent protein (VIC) dyes to detect mutant and wild-type *EGFR* alleles, respectively. The customized primers and probes were synthesized by Life Technologies (Thermo Fisher Scientific Inc., Boston, MA, USA). To evaluate the sensitivity of the T790M detection assay, DNA from NCI-H1975 cells, which harbor the T790M mutation, was serially diluted with reference human genomic DNA to achieve decreasing ratios from 1:1 to 1:2500 of the T790M mutant allele versus the wild-type allele. The final 20 μL of the TaqMan PCR mixture contained 1× ddPCR Master Mix (Bio-Rad Laboratories, Hercules, CA, USA), 900 nM of each primer, 450 nM of each probe, and 50 ng of DNA template. A maximum of 20,000 droplets could be generated from each sample for PCR. The thermal cycling conditions for the T790M detection assay comprised a 10-min incubation at 95 °C followed by 45 cycles at 95 °C for 15 s and 60 °C for 1 min, followed by a hold step at 4 °C. Analysis of ddPCR data for allele calling was performed with Quanta Soft software version 1.3.2.0 (Bio-Rad). Reference human genomic DNA (Catalog No. G1471, Promega, Wisconsin, USA) was routinely included as a negative control and used to determine the cut-off value for allele calling. Considering that single, non-specific droplets were occasionally found in the positive area, the presence of at least two droplets with the FAM signal was defined as a positive signal for the mutation. After the establishment of the ddPCR protocol, 7.3 µL of plasma cell-free DNA was added to the reaction mixture described above. The number of positive droplets and sample input followed the Poisson distribution. Plasma DNA input per reaction (*I*) was calculated with the following equation: $I\left( {{\text{copies}}/{\text{reaction}}} \right) = - { \ln }\left( { 1- {\text{p}}} \right)/{\text{V}}\; \times \; 1000\; \times \; 20$, where p represents the fraction of positive droplets, V represents the volume of each droplet (0.91 nL), and *I* represents the total number of copies of *EGFR*-mutant and wild-type DNA templates (corresponding to FAM and VIC signals, respectively).

The protocol for PCR was the same as that mentioned above. Four reference human genomic DNA samples were used as negative controls. Two positive controls with a 1:2500 ratio of mutant allele to wild-type allele and two non-template controls (NTC) were included in each run. The samples were considered positive for target mutations when they contained at least 2 droplets positive for the FAM signal. The fraction of *EGFR* T790M mutation (F1) was calculated as follows:${\text{F1}} = {\text{I}}\left( {\text{FAM}} \right)/\left[ {{\text{I}}\left( {\text{FAM}} \right) \, + {\text{ I}}\left( {\text{VIC}} \right)} \right]$. We defined a sample as ddPCR^T790M+^ when it contained at least 2 droplets positive for the FAM signal.

### Statistical analysis

Continuous data with a normal distribution were compared between two groups using Student’s *t* tests. Wilcoxon two-sample tests were performed when continuous data did not follow a normal distribution. Categorical data between two groups were compared using the Chi square or Fisher’s exact test. Concordance of T790M mutational status in ctDNA detected by ddPCR and ARMS assays was compared by McNemar’s test. PFS curves were constructed using the Kaplan–Meier method and compared using the log-rank test. Unconditional multiple logistic regression was performed to estimate risk factors of radical metastasis, and variables included *EGFR* mutation, age, smoking history, Eastern Cooperative Oncology Group (ECOG) score, therapy before TKI administration, sex and tumor pathology. OS was calculated from the time of development of TKI resistance to the time of death for any reason or last follow-up. Significance in all analyses was assessed at *P *< 0.05. All tests were two tailed. All analyses were performed using SAS software (version 9.3, SAS Institute, Cary, NC, USA).

## Results

### Patient characteristics

**Fig. 1:**
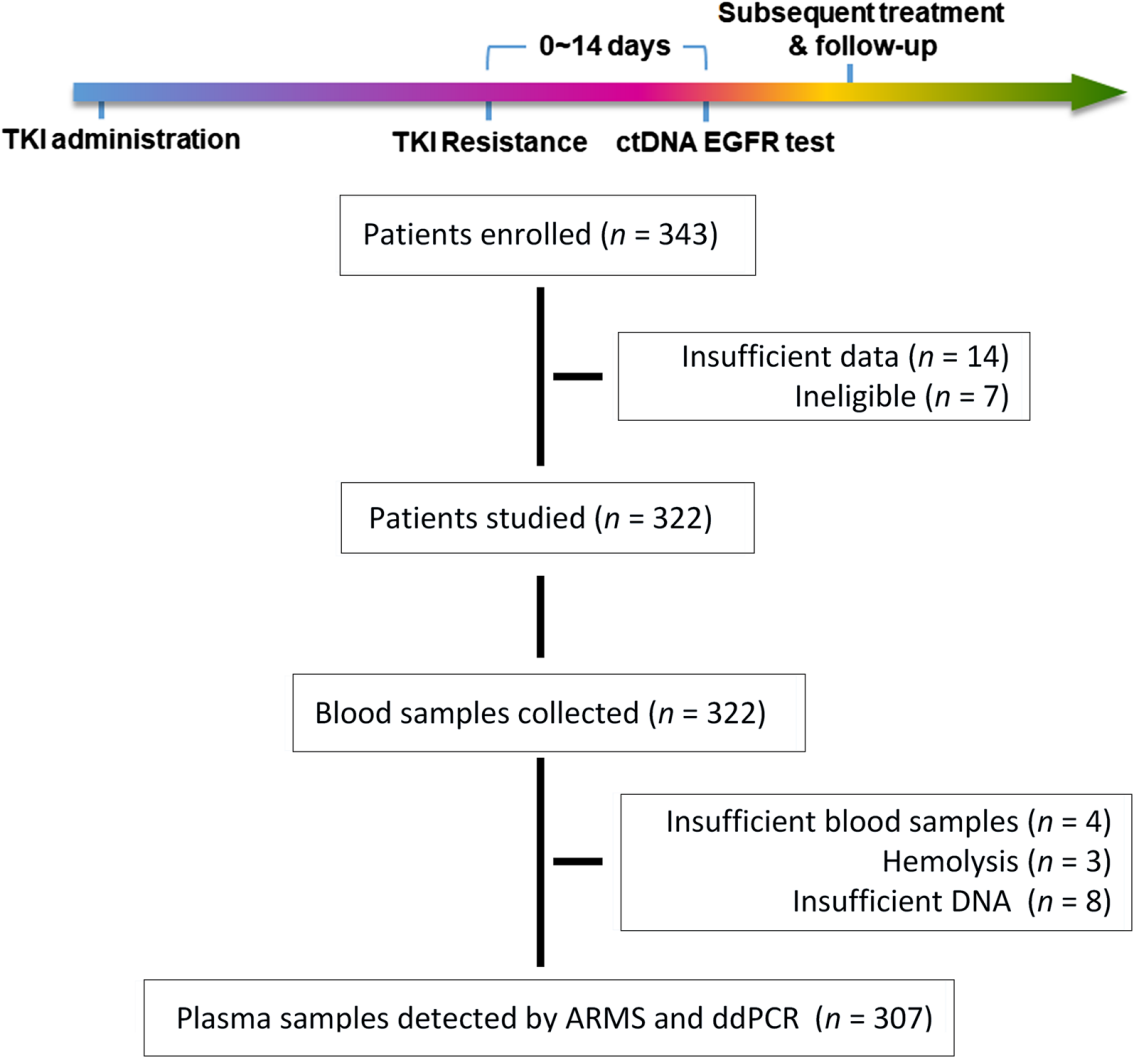
The scheme of the clinical trial design. *TKI* tyrosine kinase inhibitor, *ctDNA* circulating tumor DNA, *EGFR* epidermal growth factor receptor, *ARMS* amplification refractory mutation system, *ddPCR* droplet digital PCR

In total, 307 patients (44.0% males and 56.0% females) with advanced or recurrent NSCLC who had progressed during EGFR-TKI treatment with gefitinib, erlotinib or icotinib between March 2015 and March 2016 were consecutively enrolled in the present study. The median age of the patients was 63 years (range 32–89 years). Approximately 74.6% of the patients did not have a history of smoking. The type of NSCLC was predominantly adenocarcinoma (97.1%), and 76.5% of patients were diagnosed with stage IV disease. Moreover, 53.1% of the patients had *EGFR* 19del; 39.4% had the L858R mutation. Response to EGFR-TKI treatment was as follows: 32.9% stable disease (SD), 58.6% partial response (PR) and 8.5% complete response (CR) (Table 1). Figure 1 shows the scheme of the clinical trial design. The median follow-up time was 11 months (range 2–22 months).

**Table 1:** Clinical characteristics of non-small cell lung cancer patients with different failure sites

Factor	Total [cases (%)]	CF [cases (%)]	BF [cases (%)]	OF [cases (%)]	P
Total	307	192	32	83	
Gender					0.010
Male	135 (44.0)	90 (46.9)	19 (59.4)	26 (31.3)	
Female	172 (56.0)	102 (53.1)	13 (40.6)	57 (68.7)	
Smoking history					0.242
Never	229 (74.6)	145 (75.5)	20 (62.5)	64 (77.1)	
Former/current	78 (25.4)	47 (24.5)	12 (37.5)	19 (22.9)	
Pathology					0.776
Adenocarcinoma	298 (97.1)	186 (96.9)	31 (96.9)	81 (97.6)	
Squamous	4 (1.3)	2 (1.0)	1 (3.1)	1 (1.2)	
Adenosquamous	5 (1.6)	4 (2.1)	0 (0)	1 (1.2)	
Tumor stage					NA
IIIA	52 (16.9)	51 (26.6)	0 (0)	0 (0)	
IIIB	20 (6.5)	19 (9.9)	0 (0)	0 (0)	
IV	235 (76.5)	122 (63.5)	32 (100)	83 (100)	
Initial *EGFR* mutation					0.656
19del	163 (53.1)	106 (55.2)	18 (56.3)	39 (47.0)	
L858R	121 (39.4)	74 (38.5)	11 (34.4)	36 (43.4)	
Rare mutation	23 (7.5)	12 (6.3)	3 (9.4)	8 (9.6)	
Response					0.023
SD	101 (32.9)	63 (32.8)	15 (46.9)	23 (27.7)	
PR	180 (58.6)	116 (60.4)	17 (53.1)	47 (56.6)	
CR	26 (8.5)	13 (6.8)	0 (0)	13 (15.7)	

CF, progressive disease limited to the chest in lung/pleural tissues and lymph nodes, with no evidence of progression beyond the chest; BF, progressive disease in a previously existing site or a new site of metastatic disease in the brain, with no evidence of extracranial progression; OF, progressive disease in other distant sites or multiple sites including the chest and intracranial region

*EGFR* epidermal growth factor receptor, *CR* complete response, *PR* partial response, *SD* stable disease

### *EGFR* mutation profiles in TKI-resistant ctDNA samples by ARMS

**Fig. 2:**
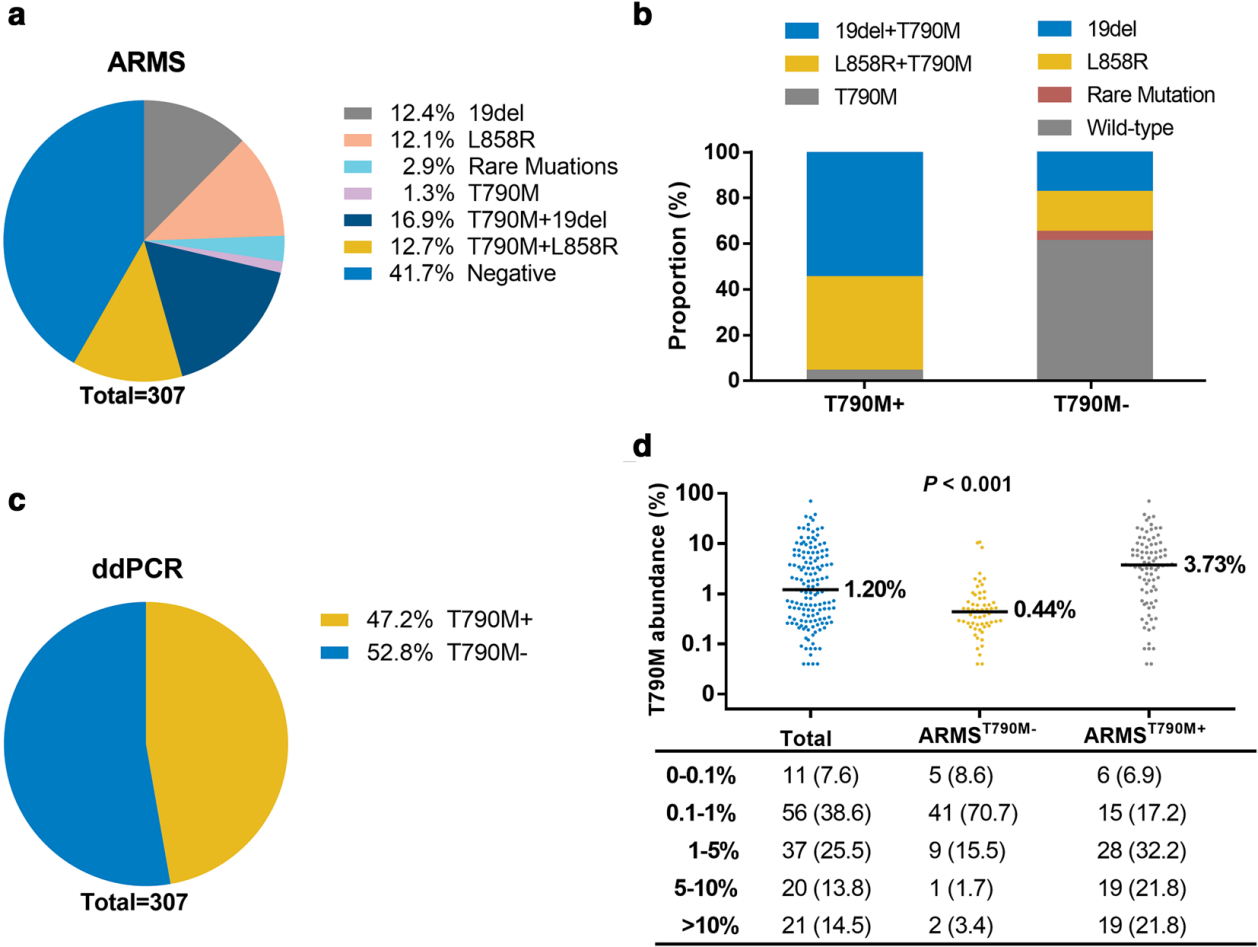
*EGFR* mutations in ctDNA samples from patients with TKI resistance. **a** *EGFR* mutation profiles determined by ARMS. **b** *EGFR* mutation profiles in T790M+ and T790M− groups assessed by ARMS. **c** T790M mutation determined by ddPCR. **d** Scatter plot of the T790M abundance in the groups according to the T790M status by ARMS (top) and the number of patients with different abundance of T790M (bottom). *TKI* tyrosine kinase inhibitor, *ctDNA* circulating tumor DNA, *EGFR* epidermal growth factor receptor, *ARMS* amplification refractory mutation system, *ddPCR* droplet digital PCR

A common reason for disease progression during EGFR-TKI treatment is the acquisition of secondary mutations such as T790M. To gain insight into T790M mutation in patients with failure at different sites, we used ARMS and ddPCR to detect possible *EGFR* mutations, including T790M, in ctDNA samples isolated from TKI-resistant patients. ARMS detected T790M mutation (ARMS^T790M+^) in 95 (30.9%) samples (Fig. 2a). In total, 84 (27.4%) samples had original *EGFR* activating mutations (19del, L858R and other rare mutations) that had been detected in primary tumor specimens before TKI administration, whereas 128 (41.7%) samples exhibited wild-type *EGFR* (Fig. 2a). Among the 95 samples with T790M mutation, 52 (54.7%) had 19del and T790M mutation, and 39 (41.1%) had L858R and T790M mutation; single T790M mutation was detected in only 4 (4.2%) samples (Fig. 2b). For T790M− samples, 128 (60.4%), 38 (17.9%), and 37 (17.5%) samples had wild-type *EGFR*, single 19del, and single L858R mutation, respectively (Fig. 2b).

### Development of a ddPCR assay for T790M mutation in TKI-resistant ctDNA samples

ddPCR was used in the present study to specifically detect and quantify ctDNA with *EGFR* T790M mutation. In total, 145 (47.2%) samples had a detectable T790M mutation (termed ddPCR^T790M+^); 162 (52.8%) samples were negative for T790M (termed ddPCR^T790M−^) (Fig. 2c). The median abundance of T790M was 1.20% (range 0.04%–70.30%) for 145 ddPCR^T790M+^ samples, with 0.44% (range 0.04%–10.77%) for ddPCR ^T790M+^ ARMS^T790M−^ samples and 3.73% (range 0.04%–70.30%) for ddPCR^T790M+^ ARMS^T790M+^ samples (Fig. 2d). Of note, ddPCR detected a significantly greater abundance of T790M mutation in the ARMS^T790M+^ group than in the ARMS^T790M−^ group. In addition, among 145 ddPCR^T790M+^ patients, T790M abundance for 11 patients (7.6%) was within the range of 0–0.1%, 56 (38.6%) within 0.1–1%, 37 (25.5%) within 1–5%, 20 (13.8%) within 5–10%, and 21 (14.5%) above 10% (Fig. 2d).

The overall concordance of ARMS and ddPCR was 78.5% (241/307) (with a kappa value of 0.561), indicating that both methods were highly consistent. Relative to the corresponding sensitivity and specificity of ARMS, ddPCR showed a sensitivity of 91.6% (95% confidence interval (CI) 86–96.7%) and a specificity of 72.6% (95% CI 67.0–78.4%) in detecting T790M mutation (Table 2).

**Table 2:** Comparison of T790M mutation, as detected by ddPCR and ARMS

ARMS	ddPCR		Total
	T790M+	T790M−	
T790M+	87	8	95
T790M−	58	154	212
Total	145	162	307

*ARMS* amplification refractory mutation system, *ddPCR* droplet digital PCR

### Association between T790M mutation and disease failure site

To assess correlations between T790M mutation and failure site, the 307 patients were categorized into three groups: 192 (62.5%) into the CF group, 32 (10.4%) into the BF group, and 83 (27.0%) into the OF group. The clinical characteristics of these three groups are shown in Table 1. The median ages were 64, 62, and 63 years old in patients with CF, BF, and OF groups, respectively. There was no significant difference in age, smoking history, pathology or initial *EGFR* mutation type among the three groups. However, the BF group was predominantly male and the OF group predominantly female (*P *= 0.01). TKI response also differed, with patients in the BF group having the highest percentage of SD but a lower rate of PR and CR than did patients in the CF and OF groups (*P *= 0.023) (Table 1).

**Fig. 3:**
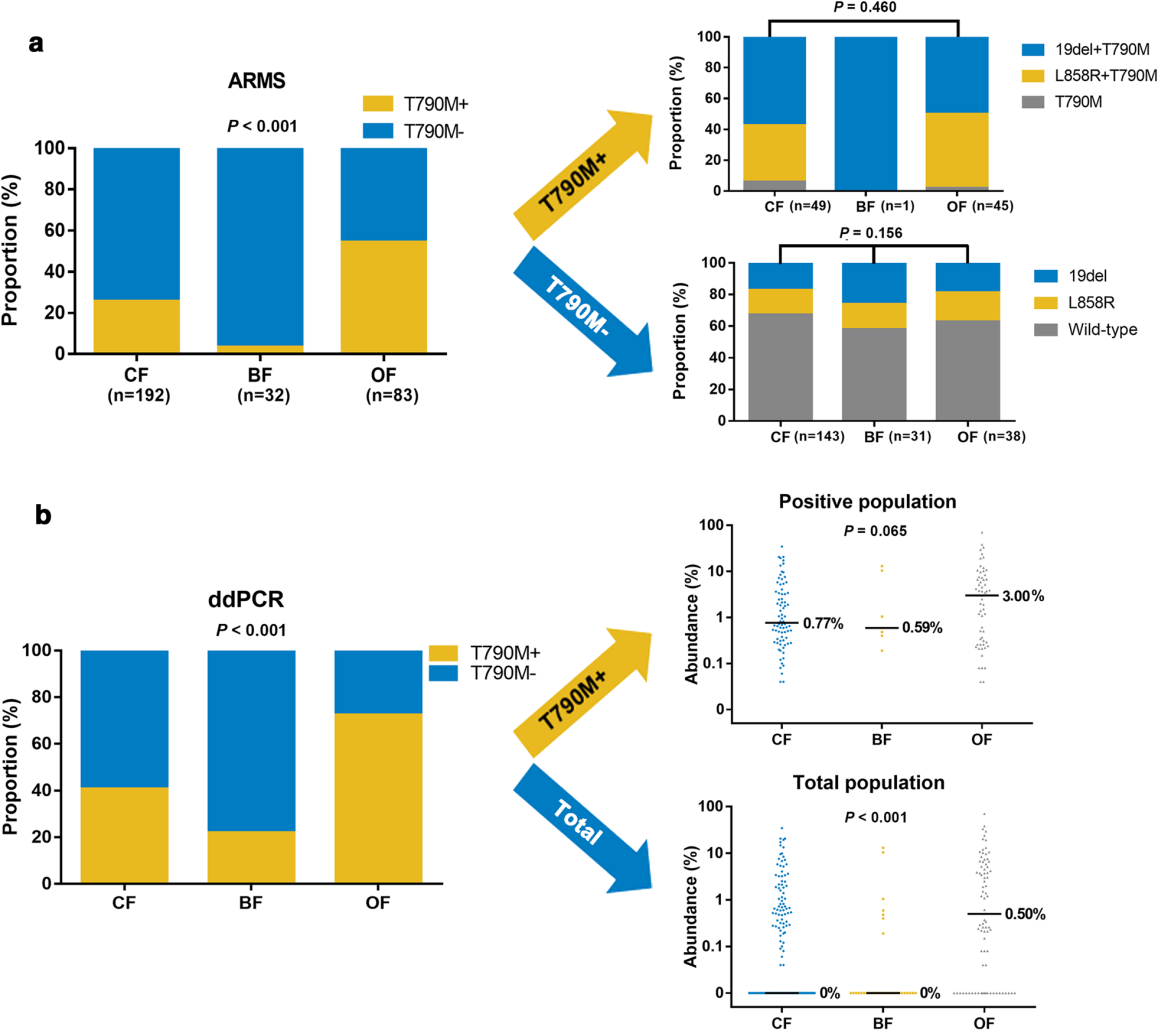
Distribution of *EGFR* mutations between different failure sites. **a** The proportion of ARMS^T790M+^ and ARMS^T790M−^ in different failure sites (left). The proportion of ARMS^19del+T790M^, ARMS^L858R+T790M^, and ARMS^T790M+^ in different failure sites (up-right). The distribution of ARMS^wild-type^, ARMS^19del^, and ARMS^L858R^ in different failure sites (down-right). **b** The proportion of ddPCR^T790M+^ and ddPCR^T790M−^ (left), as well as the abundance of T790M in patients with detectable T790M mutation (up-right) and in total patients (down-right). *ARMS* amplification refractory mutation system, *ddPCR* droplet digital PCR

Overall, 49 (25.5%) of 192 patients in the CF group and 45 (54.2%) of 83 patients in the OF group were ARMS^T790M+^, whereas only 1 (3.1%) of 32 patients in the BF group was ARMS^T790M+^ (*P *< 0.001) (Fig. 3a, left). According to ddPCR, a similar distribution of T790M mutation was observed among the three groups (Fig. 3b): the proportion of ddPCR^T790M+^ patients was 40.6% (78 of 192) for the CF group, 21.9% (7 of 32) for the BF group, and 72.3% (60 of 83) for the OF group (*P *< 0.001) (Fig. 3b, left).

We next explored the distribution of different subtypes of T790M mutation (T790M, 19del + T790M, L858R + T790M) in the three groups (Fig. 3a, right). However, no significant difference in the distribution of the different mutational subtypes was observed for either ARMS^T790M+^ (*P *= 0.460) or ARMS^T790M−^ (*P *= 0.156) patients (Fig. 3a, right).

Furthermore, the median abundance of T790M mutation in ddPCR^T790M+^ patients was 0.77, 0.59, and 3% for the CF, BF, and OF groups, respectively (*P *= 0.065) (Fig. 3b, right). Of note, pair-wise comparison showed that the difference between the OF and CF groups was significant (*P *= 0.047) (Fig. 3b, right). Nonetheless, when all patients were included in the analysis, the median abundance of T790M mutation was significantly different among the groups, with 0, 0 and 0.5% for the CF, BF, and OF groups, respectively (*P *< 0.001) (Fig. 3b, right).

### Effect of T790M mutation in plasma on clinical prognosis after the development of resistance to first-generation EGFR-TKI

To assess the effect of the presence of T790M mutation in plasma samples on prognosis after first-generation TKI resistance development, we followed up the patients every 3 months via telephone until April 2017; we recorded data for 278 patients, as 29 were lost to follow-up. As shown in Table 3, most patients received subsequent treatment. The T790M− patients in all three groups were likely to continue receiving the original TKI, whereas the T790M+ patients in the OF group were most likely to receive AZD9291.

**Table 3:** Subsequent treatment after first-generation epidermal growth factor receptor-tyrosine kinase inhibitor resistance

Group	Total (cases)	Continuation of TKI [cases (%)]	AZD9291 [cases (%)]	Chemo ± RT [cases (%)]	TKI plus chemo/RT [cases (%)]	BSC [cases (%)]
CF						
T790M+	72	23 (31.9)	28 (38.9)	10 (13.9)	4 (5.6)	7 (9.7)
T790M−	103	41 (39.8)	23 (22.3)	21 (20.4)	9 (8.7)	9 (8.7)
Total	175	64 (36.6)	51 (29.1)	31 (17.7)	13 (7.4)	16 (9.1)
BF						
T790M+	6	1 (16.7)	1 (16.7)	0 (0)	0 (0)	4 (66.7)
T790M−	21	9 (42.9)	3 (14.3)	2 (9.5)	2 (9.5)	5 (23.8)
Total	27	10 (37.0)	4 (14.8)	2 (7.4)	2 (7.4)	9 (33.3)
OF						
T790M+	53	10 (18.9)	22 (41.5)	11 (20.8)	4 (7.5)	6 (11.3)
T790M−	23	7 (30.4)	8 (34.8)	3 (13)	1 (4.3)	4 (17.4)
Total	76	17 (22.4)	30 (39.5)	14 (18.4)	5 (6.6)	10 (13.2)

CF, progressive disease limited to the chest in lung/pleural tissues and lymph nodes, with no evidence of progression beyond the chest; BF, progressive disease in a previously existing site or a new site of metastatic disease in the brain, with no evidence of extracranial progression; OF, progressive disease in other distant sites or multiple sites including the chest and intracranial region

*TKI* tyrosine kinase inhibitor, *Chemo* chemotherapy, *RT* radiotherapy, *BSC* best supportive care

**Fig. 4:**
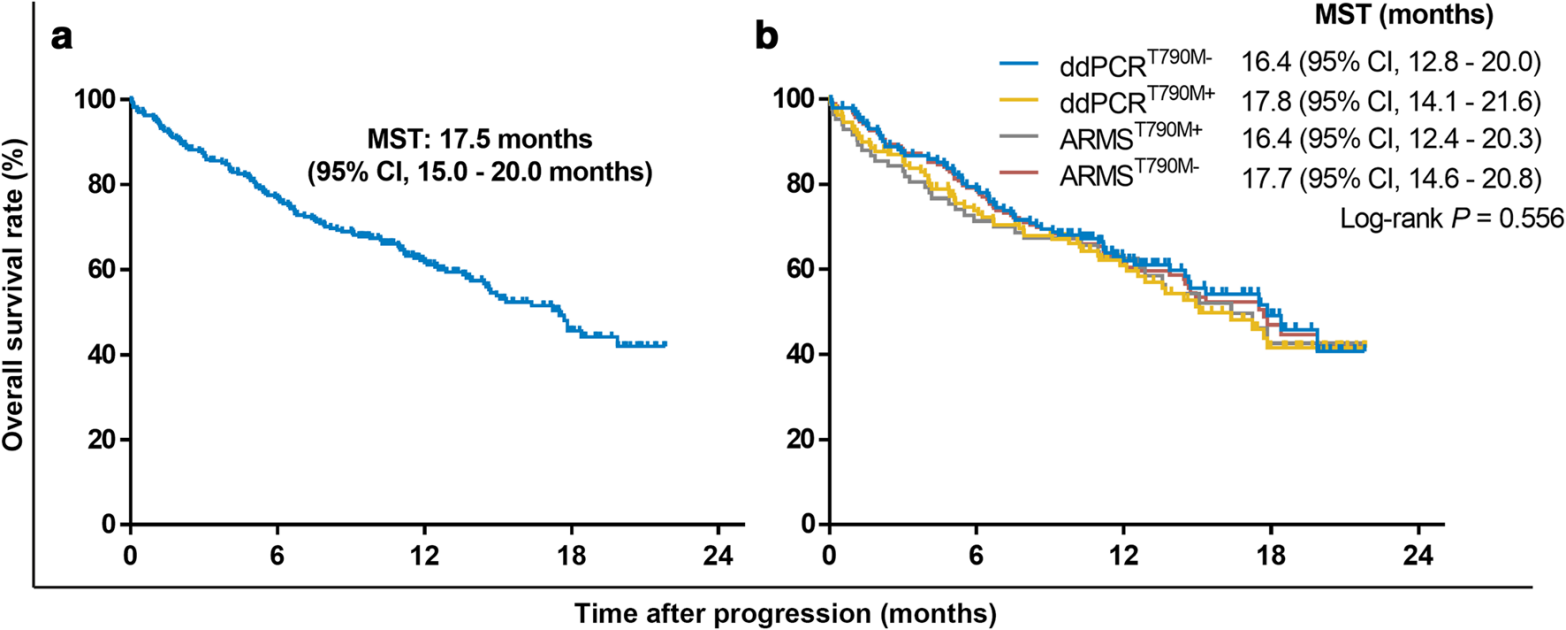
Survival curves of patients with different T790M mutations. **a** Survival curves after TKI failure in 307 patients. **b** OS curves for patients with different T790M status, as assessed by ARMS and ddPCR. *MST* median survival time, *ARMS* amplification refractory mutation system, *ddPCR* droplet digital PCR

**Fig. 5:**
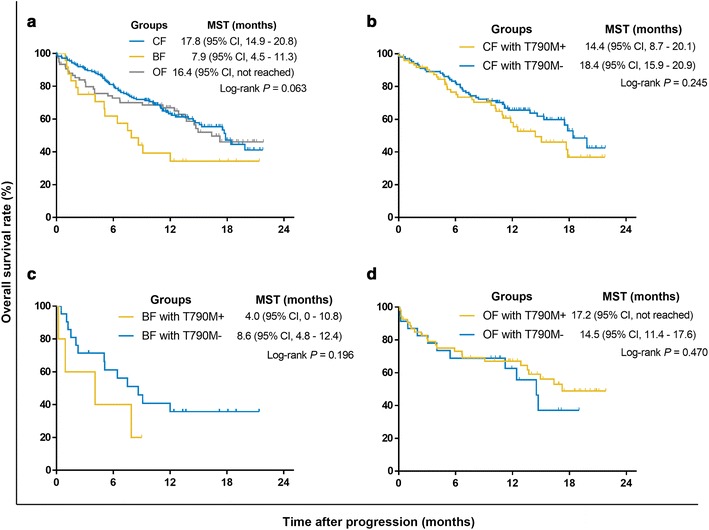
Survival curves of patients with different failure sites. **a** Survival curves categorized by failure site. **b** Survival curves of T790M+ and T790M− CF patients. **c** Survival curves of T790M+ and T790M− BF patients. **d** Survival curves of T790M+ and T790M− OF patients. CF, progressive disease limited to the chest in lung/pleural tissues and lymph nodes, with no evidence of progression beyond the chest; BF, progressive disease in a previously existing site or a new site of metastatic disease in the brain, with no evidence of extracranial progression; OF, progressive disease in other distant sites or multiple sites including the chest and intracranial region. T790M mutation was detected by ddPCR. *MST* median survival time, *ARMS* amplification refractory mutation system, *ddPCR* droplet digital PCR

**Fig. 6:**
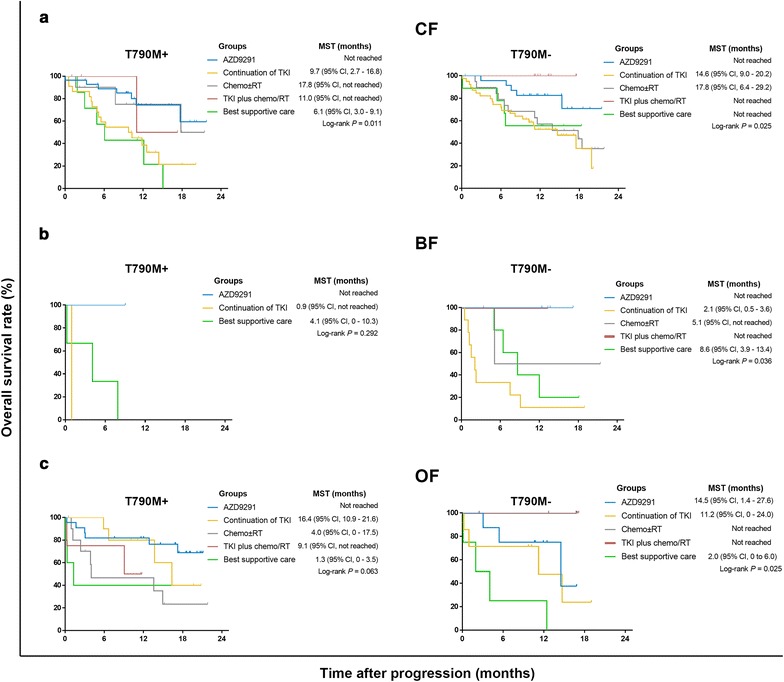
Survival curves of patients receiving different subsequent treatments. **a** Curve for T790M+ and T790M− CF patients. **b** Curve for T790M+ and T790M− BF patients. **c** Curve for T790M+ and T790M− OF patients. CF, progressive disease limited to the chest in lung/pleural tissues and lymph nodes, with no evidence of progression beyond the chest; BF, progressive disease in a previously existing site or a new site of metastatic disease in the brain, with no evidence of extracranial progression; OF, progressive disease in other distant sites or multiple sites including the chest and intracranial region. T790M mutation was detected by ddPCR

The median survival time after progression on TKIs was 17.5 months (95% CI 15–20 months) (Fig. 4a). Patients with/without T790M mutation had similar survival durations (Fig. 4b). Although patients in the CF and OF groups had longer survival times than did patients in the BF group, there was no significant difference in survival among the three groups according to T790M mutational status (Fig. 5). In the CF group, T790M+ patients undergoing AZD9291 treatment had the best survival duration (median survival time not reached), followed by patients treated with chemotherapy ± radiotherapy (chemo ± RT) and TKI + chemo/RT (17.8 and 11.0 months, respectively). Continuation of TKIs and best supportive care (BSC) yielded the shortest survival duration (9.7 and 6.1 months, respectively) (Fig. 6a). In T790M− patients in the CF group, TKI + chemo/RT conferred the longest survival duration (median survival time not reached), and the continuation of TKIs yielded the shortest survival duration (14.6 months) (Fig. 6a). Due to the small sample size, we did not observe a significant difference among T790M+ patients in the BF group (Fig. 6b). In the T790M− patients in the BF group, AZD9291 and TKI + chemo/RT resulted in better survival durations than did the other treatments (Fig. 6b). In T790M+ patients in the OF group, AZD9291 was the best option, whereas the median survival duration for the chemo ± RT group and BSC was merely 4.0 and 1.3 months, respectively (Fig. 6c). In the T790M− patients in the OF group, TKI + chemo/RT and chemo ± RT resulted in the longest survival durations (Fig. 6c). In total, 85 patients (51 with T790M mutation) received AZD9291 after progressing on first-generation EGFR-TKIs. Two patients were lost to follow-up. Furthermore, 49 T790M+ patients had response records. The disease control rates of T790M+ patients after AZD9291 administration were 92.3% (24/26), 100% (1/1), and 77.3% (17/22) for the CF, BF, and OF groups, respectively.

## Discussion

In the present study, we used ARMS and ddPCR to detect possible T790M mutation in plasma ctDNA collected from patients after the failure of EGFR-TKIs. The results demonstrate a strong concordance between ARMS and ddPCR for the detection of T790M mutation in plasma ctDNA. Because ddPCR also possesses high sensitivity and specificity, we suggest that ddPCR is a useful tool for determining T790M mutation and for quantifying both the frequency and abundance of T790M mutation in plasma ctDNA. Based on our ddPCR results, 47.2% of patients had detectable T790M mutation, similar to the findings of previous studies [22, 34, 35]. The relatively low abundance (median abundance of 1.2%) indicates the complexity of detecting T790M mutation in plasma.

Interestingly, we identified a significant association between the plasma T790M mutational status and failure sites in the present study. We found that patients in the OF group had a higher frequency of T790M mutation and a higher mutational abundance than did patients in the CF and BF groups, which was consistent with the results reported by Thress et al. [21]. Theoretically, ctDNA levels are generally correlated with the tumor burden; more metastatic tumors shed more DNA into the bloodstream, resulting in higher levels of tumor-derived DNA. Consistent with this hypothesis, a high rate of T790M mutation was detected in patients in the OF group. However, tumor burden alone was not sufficient to explain the higher T790M abundance in patients in the OF group compared to those in the CF and BF groups because T790M abundance is calculated from the ratio of T790M mutation to total *EGFR* copy number. A higher capacity for invasion and migration in cancer cells with T790M mutation may have led to the higher abundance of these mutations in patients in the OF group.

Brain metastasis (BM) accounts for approximately 50% of NSCLC recurrence after TKI treatment [36]. First-generation EGFR-TKIs commonly have a low capacity to penetrate the blood brain barrier (BBB) (0.6–1.3% for gefitinib and 2.8–4.4% for erlotinib). TKIs are also a substrate of P-glycoprotein, an efflux pump located on the membrane of endothelial cells [37, 38]. Therefore, intracranial failure of EGFR-TKIs is mainly considered a pharmacokinetic limitation rather than emergence of T790M mutation [39–42]. Zhao et al. [43] analyzed paired plasma and cerebrospinal fluid (CSF) samples from seven NSCLC patients with CNS metastases after failure with first-generation EGFR-TKIs and found that all harbored EGFR-TKI-sensitive mutations in CSF but that only one harbored T790M mutation in CSF. Exposure of gefitinib for all patients was significantly low in CSF, with a mean CSF/plasma mutational rate of 1.8%. Another study identified T790M mutation in 7 of 9 patients with extracranial lesions using circulating tumor cells (CTCs) from CSF but in only 1 of 14 patients using CSF samples [44]. In the present study, the lowest frequency and abundance of T790M mutation in plasma ctDNA was detected in patients in the BF group. In addition, several other studies have reported a low incidence of T790M mutation in CSF of a small number of BM patients [45, 46]. Therefore, we suggest that T790M mutation, one of the predominant resistance mechanisms, might not contribute substantially to BM. Future studies using brain tumor tissues might provide a better understanding of the role of T790M mutation in patients developing BM during EGFR-TKI therapy.

Several retrospective studies have reported controversial results regarding the prognostic value of acquired T790M mutation. Studies using tumor re-biopsies showed that NSCLC patients with acquired T790M mutation had prolonged survival durations; however, several studies using ctDNA-based mutational analysis demonstrated that ctDNA T790M+ patients had significantly short OS [15, 18–20]. Zheng et al. [22] reported that among patients receiving TKI treatment as a 2nd-line or later treatment, those with detectable T790M mutation in ctDNA had poor survival. In our study, ddPCR^T790M−^ patients had a longer median PFS on first-generation EGFR-TKIs than did ddPCR^T790M+^ patients; a similar trend was also observed by ARMS, indicating that the tumors that had progressed in patients with plasma ctDNA-detectable T790M mutation were highly aggressive. We also observed longer survival in T790M− patients than in T790M+ patients based on the time of initial TKI administration (data not shown). However, when we calculated survival from the initial development of resistance to TKIs, we failed to observe any difference between; these findings were similar to the results of a study by Zhang et al. [47].

Furthermore, by following up the patients every 3 months, we assessed the effect of T790M mutation on subsequent clinical prognosis after progression on first-generation TKI treatment. Different lesions from patients with advanced NSCLC including recurrent or enlarged tumors or metastases requiring different treatments. The median survival time after progression on TKIs was 17.5 months, and the five types of treatments used in patients were continuation of the original TKI, AZD9291, chemo ± radiation, TKI + chemo/RT and BSC. For each subgroup of T790M+ patients in the CF, BF and OF groups, AZD9291 treatment appeared to be the best option, conferring the longest survival duration. These data are consistent with the findings of AURA trials, reporting a higher response rate to AZD9291 (61%) among patients with T790M mutation than among patients without this mutation (21%), with a median PFS of 9.6 months in T790M+ patients and 2.8 months in T790M− patients [48]. Moreover, in our study, the survival of patients treated with chemo ± RT and TKI + chemo/RT was acceptable in the T790M + CF group but not in the T790M + OF group (4.0 to 9.1 months). Patients receiving concurrent chemoradiation or TKI + RT had long survival durations, even those in the T790M− CF group. These data indicate that local treatment may have contributed to clinical benefit when the disease was localized, though this may not have been the case for patients with extensive metastasis. TKI + chemo/RT yielded the best survival in T790M− patients of the CF and OF groups. In the IMPRESS study, concurrent chemotherapy combined with TKIs after PD did not confer any benefit in the total population, whereas TKIs in combination with chemotherapy did provide a clinical benefit for patients with T790M− plasma ctDNA [49]. Nevertheless, further investigation is required to determine whether TKI + chemo can benefit T790M− patients after resistance to first-generation EGFR-TKIs. In the present study, patients, both T790M+ and T790M− in the BF group, treated with AZD9291 had the best survival. Considering the better penetration of AZD9291, as reported in the BLOOM study, and its great potency for targeting both sensitive and resistant mutant NSCLC cells [50], the reason for the excellent efficacy of AZD9291 in both T790M+ and T790M− BF groups is rather clear. Thus, both T790M mutational status and failure site strongly influence treatment selection and therapeutic effect.

This is a large-scale multi-institutional study to demonstrate correlation between disease failure sites and clinical outcomes after TKI therapy with the frequency/abundance of T790M mutation in ctDNA. Nevertheless, several limitations exist. First, molecular profiling was not simultaneously performed on matched tissue biopsies and blood samples after disease progression, and such analysis may reveal more direct correlation and concordance between both sets of samples, thereby enabling a better understanding of their distinct clinical features. Second, dynamic changes in T790M mutation in ctDNA were not monitored during disease progression, which may have been helpful in following disease progression among the three groups based on failure site. Third, only a single *EGFR* mutation was investigated in the present study due to limitations in one-time ddPCR and ARMS. Further studies to overcome these limitations are needed in the future.

## Conclusion

Our prospective study demonstrated an important clinical value of T790M mutation in the ctDNA of patients who progressed on EGFR-TKI therapy. We show that ddPCR has a higher sensitivity than does ARMS. Our study revealed that the T790M mutational frequency and abundance are correlated with disease failure sites. Patients in the OF group were more likely to harbor T790M mutation than were those in the CF and BF groups. In addition, the T790M mutational status in ctDNA has a substantial influence on clinical prognosis based on subsequent treatment in each subgroup stratified by different failure sites. AZD9291 was the most frequent choice for T790M+ patients and yielded the longest survival after resistance to first-generation EGFR-TKIs. These data suggest that different therapeutic strategies should be considered for TKI-resistant patients depending on their T790M mutational status and disease failure site.

## Additional file

**Additional file 1: Figure S1.** Design of the assay for detection of *EGFR* T790M mutations. (A) FAM- and VIC-labeled probes were designed to target mutant and wild-type EGFR alleles, respectively. (B) Sequence information of the primers and probes for the T790M ddPCR assay. (C) Selective sensitivity of the assay for T790M mutation. Up to 1:2,500 dilution of mutant to wild-type *EGFR* alleles; at least two positive droplets were stably detected by the ddPCR assay. The numbers shown in the positive area are the amounts of *EGFR* mutant allele-positive droplets by ddPCR. Mt, mutant allele; Wt, wild-type allele; ddPCR, droplet digital PCR.

## Abbreviations

ARMS: amplification refractory mutation system
CNS: central nervous system
CR: complete disease
ctDNA: circulating tumor DNA
ddPCR: droplet digital PCR
ECOG: Eastern Cooperative Oncology Group
EGFR: epidermal growth factor receptor
NSCLC: non-small cell lung cancer
TKI: tyrosine kinase inhibitor
ORR: objective response rate
OS: overall survival
PD: progressive disease
PFS: progression-free survival
PR: partial disease
SD: stable disease
