# Supplementary figures and images for: Epidermal growth factor receptor (EGFR) T790M mutation identified in plasma indicates failure sites and predicts clinical prognosis in non-small cell lung cancer progression during first-generation tyrosine kinase inhibitor therapy: a prospective observational study
Source: Cancer Commun (Lond). 2018 May 22;38:28. doi: 10.1186/s40880-018-0303-2 (PMC5993134; doi:10.1186/s40880-018-0303-2)

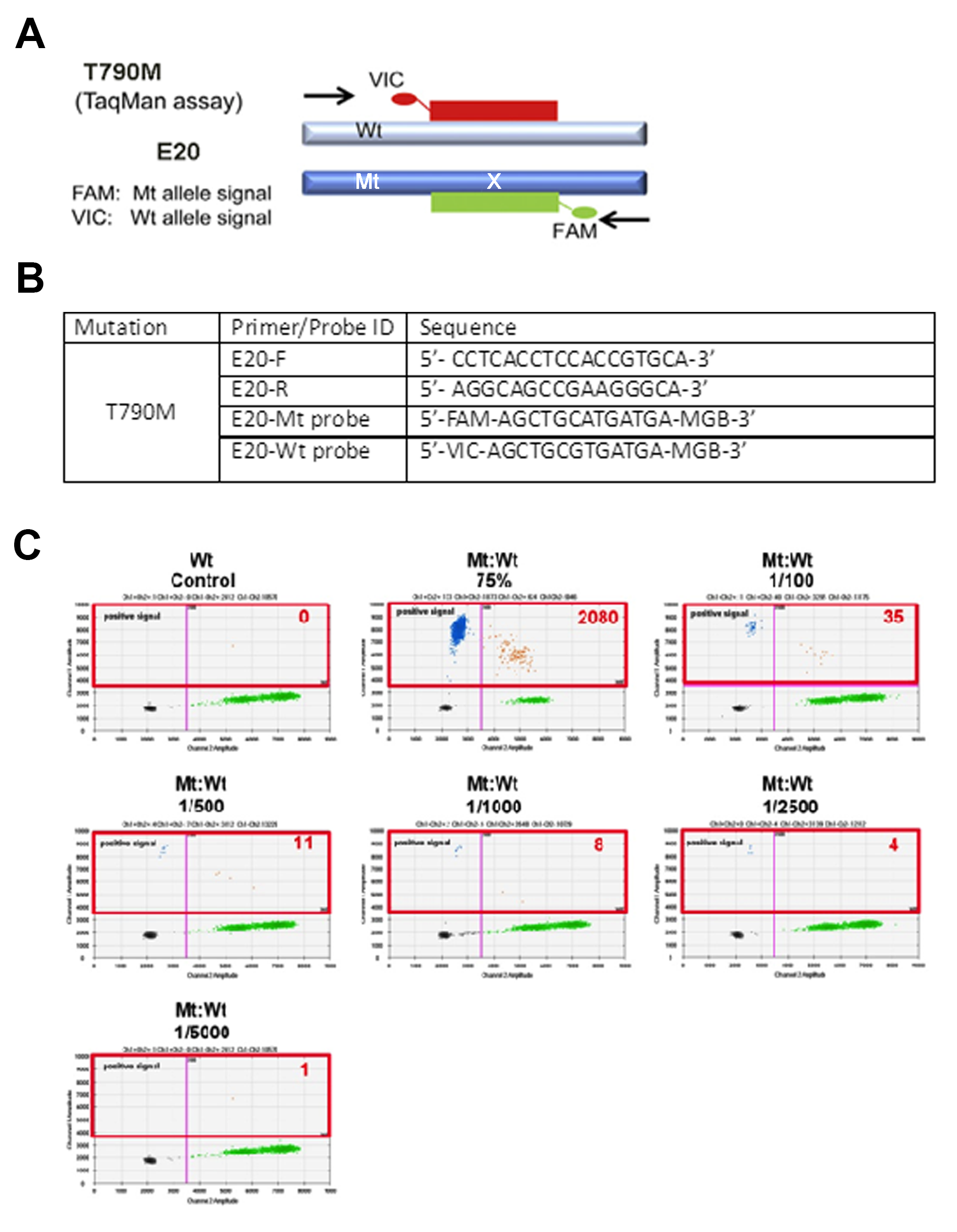

Supplement: Supplementary file 1 — Additional file 1: Figure S1. Design of the assay for detection of EGFR T790M mutations. (A) FAM- and VIC-labeled probes were designed to target mutant and wild-type EGFR alleles, respectively. (B) Sequence information of the primers and probes for the T790M ddPCR assay. (C) Selective sensitivity of the assay for T790M mutation. Up to 1:2,500 dilution of mutant to wild-type EGFR alleles; at least two positive droplets were stably detected by the ddPCR assay. The numbers shown in the positive area are the amounts of EGFR mutant allele-positive droplets by ddPCR. Mt, mutant allele; Wt, wild-type allele; ddPCR, droplet digital PCR. [file 40880_2018_303_MOESM1_ESM.tif]
